# The recruitment of global language inhibitory control and cognitive-general control mechanisms in comprehending language switches: Evidence from eye movements

**DOI:** 10.1017/S1366728924000567

**Published:** 2024-12-04

**Authors:** Ana I. Schwartz, Joseph Negron, Colin Scholl

**Affiliations:** Department of Psychology, The University of Texas at El Paso, El Paso, TX, USA

## Abstract

Prominent models of the bilingual lexicon do not allow for language – wide inhibition or any effect of general cognitive control on the activation of words within the lexicon. We report evidence that global language inhibitory control and cognitive general control mechanisms affect lexical retrieval during comprehension. Spanish–English bilinguals read language-pure or sentences with mid-sentence switches while their eye movements were recorded. A switch cost was observed in aspects of the eye-tracking record reflecting early spread of lexical activation, as well as later measures. The switch cost was larger for L2-to-L1 switches and was not attenuated when switched words were cognates (Experiment 1). In Experiment 2, switch costs were reduced when the sentences contained a language color cue. These findings are inconsistent with the predictions of the Bilingual Interactive Activation Plus (BIA+) but support the architecture of its predecessor, the BIA. They refute the assumption that early lexical activation is impervious to nonlinguistic cues.

It is well-established that both languages of a bilingual are active irrespective of their intentions or awareness of communicating in just one. What control mechanisms are available to bilinguals that allow them to produce and comprehend in a target language in the face of continuous competition from the nontarget language? In the research on production, it is uncontroversial that language control is exerted through a combination of both reactive and proactive control mechanisms (e.g., Abutalebi & Green, 2007; Declerck & Philipp, 2015; Green, 1998).

What about comprehension? Far less research has been dedicated to this domain overall. According to the most widely tested and influential models of the bilingual mental lexicon, the Bilingual Interactive Activation Plus (BIA+) (Dijkstra & Van Heuven, 2002) model there is no language-wide control of activation. Instead, the accessibility of lexical representations within the bilingual lexicon is solely affected by transient excitatory and inhibitory activation dynamics from co-activated lexical candidates that emerge as a word is lexically retrieved. This assumption is supported by a truly impressive volume of empirical studies demonstrating that the time it takes bilinguals to recognize words in one of their languages is fundamentally influenced by their lexical form similarity across languages, even in exclusively monolingual experimental contexts (see Dijkstra, 2005; Palma & Titone, 2020; Schwartz & Van Hell, 2012 and van Assche et al., 2020 for reviews). However, studies comparing processing times of cross-language homonyms such as cognates and interlingual homographs versus controls do not speak directly to the issue of language-wide control. While these findings clearly demonstrate words across both languages are continuously available, they do not speak to potential differences in their accessibility.

A second assumption of the BIA+ is that activation dynamics within the lexicon are unaffected by nonlinguistic sources of information, such as participant expectations or response adaptations to task demands. Such information only influences post-lexical access output processes (e.g., Dijkstra, De Bruijn et al., 2000; Dijkstra, Timmermans & Schriefers, 2000). However, it is important to point out that, because of its stimulus-driven nature, written comprehension typically obviates the need for the language system to capture and respond to nonverbal cues. Although nonverbal cues may not *typically* be exploited during comprehension that does not mean that when they are afforded and informative that they *cannot* be used.

In the present study, we test the assumption that in written comprehension there is no language-wide control of activation through a language-switching paradigm. Across two experiments, we presented highly proficient, Spanish–English bilinguals with sentences that on critical trials contained a full intra-sentential switch and compared both directions of the switch. In Experiment 1, we further manipulated the cognate status of the first switched word. If there is no language-wide control of activation, then the time spent reading critical words should not be affected by whether they mark a language switch or are in the same language. Instead, reading time should solely be affected by factors known to influence lexical retrieval, such as whether the word is in the dominant (L1) or weaker language (L2) and whether it is a cognate or noncognate. Neither factor should interact with whether the word constitutes a language switch or not. If, on the other hand, there is language-wide control of activation, then we should observe a main effect of language switch, with inflated reading times for critical words that are language-switched, reflecting the need to overcome inhibition. Furthermore, if more inhibition is required to suppress the L1, the switch cost will be greater for L2-to-L1 switches relative to L1 to L2. This is precisely the opposite of what is predicted based on the current architecture of the BIA+.

In Experiment 2, we test the assumption that early phases of bilingual lexical retrieval are impervious to nonlinguistic cues by manipulating whether language is cued by font color. If bottom-up lexical activation dynamics are truly insular, then the provision of a color cue should have no bearing on processing a language switch. However, if a color cue is observed to significantly attenuate switch costs not only does this suggest that the language system can exploit nonlinguistic cues but it would also suggest the operation of cognitive general control mechanisms in the comprehension of written language.

A critical feature of the BIA+ assumptions being tested is that they pertain to the earliest phases of lexical processing, prior to the completion of retrieval. Therefore, they can only be tested with a technology, such as eye tracking that affords a continuous measure of processing with excellent temporal resolution. This allowed us to discriminate between processes occurring during the earliest phases of lexical retrieval versus later acting processes that could be attributable to post-lexical access strategies and decision processes. We next review the assumptions of the BIA+ in more detail and contrast it with its predecessor the BIA (Dijkstra & Van Heuven, 1998).

## 1. The BIA and BIA+ models of the bilingual lexicon

The BIA+ (Dijkstra & Van Heuven, 2002) and its predecessor, the BIA (Dijkstra & Van Heuven, 1998) were developed specifically as accounts of bilingual word recognition and comprehension processes. A key assumption of both of these models is that lexical representations across both of a bilingual’s languages are housed in an integrated lexicon. As such, the degree of activation of a lexical representation is a function of (1) its lexical form overlap with the incoming stimulus word and (2) excitatory and inhibitory activation dynamics produced by the set of coactivated lexical candidates within the lexicon. These dynamics are not language system wide – rather they are more “localized”, affecting only lexical representations with pertinent lexical form overlap. In this way, a word’s activation in the lexicon has nothing to do with its language membership, which in the BIA+ is represented as a nonfunctional “language tag”. This is a departure from the earlier BIA. The BIA included language nodes that collected bottom-up activation from their corresponding words and exerted language-wide inhibition of lexical representations in the competing language. These nodes allow the BIA to account for language switch costs. With each incoming word from language “A”, its corresponding language node exerts increasing inhibition of words in language “B”; thus, when there is a switch to language “B” this inhibition must be overcome, delaying processing. Another architectural assumption of the language nodes is that the more dominant L1 collects greater activation from its constituent, relatively higher frequency L1 words and thus exerts greater inhibition on the L2.

In the BIA+ (Dijkstra & Van Heuven, 2002), the language nodes no longer have this functionality. Instead, the nodes simply represent language membership information that is potentially accessed post-lexical access. This difference in architecture implies very different assumptions regarding how language control is exerted. Within the original BIA (Dijkstra & Van Heuven, 1998), language control is exerted as the language nodes exert top-down inhibition of the competing language. This control can affect the relative accessibility of words in the nontarget language, even at the earliest stages of lexical retrieval. In contrast, the BIA+ does not allow for this type of sustained control. Instead, its architecture only allows for transitory inhibition, which arises exclusively from the cohort of co-activated lexical competitors within the lexicon. Sustained control processes are only carried out by a task/decision system, which resides outside of the lexicon and have no direct effect on the activation/competition dynamics within the lexicon. Therefore, it does not modulate or have any impact on the accessibility or activation level of lexical competitors during lexical retrieval. Instead, its control is exerted exclusively post-lexical access, having an impact on decision processes and response output.

A second assumption of the BIA+ (Dijkstra & Van Heuven, 2002), which is also shared with the BIA (Dijkstra & Van Heuven, 1998), is that cognitive general control processes outside of the lexicon do not exert a direct effect on activation dynamics within the lexicon. Instead, such processes are handled by a task/decision system, which adapts to task demands by modulating decision criterion thresholds. As such, cognitive general language control mechanisms do not have an impact on the accessibility of lexical competitors within the lexicon.

## 2. Is there language-wide control of activation in comprehension?

Comprehension studies converge in observing a switch cost (see Declerck & Koch, 2023 for a review). The uniformity of switch costs across studies is particularly striking when one considers the diversity of paradigms and tasks that have been used across studies. Tasks have ranged from language-specific and language-general lexical decision (Thomas & Allport, 2000; Von Studnitz & Green, 2002), to semantic categorization (Macizo et al., 2012), to dual-task “PRP” paradigms (Hirsch et al., 2015) and to mapping sentences to pictures (Philipp & Huestegge, 2015). Although the preponderance of this evidence may seem to refute the BIA+ assumption that there is no language-wide control, there are two chief characteristics of these studies that preclude them from providing a direct test of the BIA+. First, the paradigms mostly lack the temporal sensitivity required to capture early-acting language control. The BIA+ does allow for language control dynamics to affect processing post-lexical access. Therefore, it can accommodate most of the findings listed above. Second, comprehension studies have disproportionately relied on tasks that include artificial task demands, which may heighten the involvement of task-related control processes.

Only a handful of studies have examined language switching in natural reading tasks such as sentence comprehension (Bultena et al., 2015; Hoversten & Traxler, 2020; Litcofsky & Van Hell, 2017; Moreno et al., 2002; Proverbio et al, 2004). Three of these studies employed methodologies with excellent temporal resolution such as eye tracking (Hoversten & Traxler, 2020) and EEG/ERP (Litcofsky & Van Hell, 2017; Moreno et al., 2002, Proverbio et al., 2004). Despite considerable variability in the proficiency profile of participants across these studies (ranging from highly proficient, professional translators to L1 dominant, unbalanced bilinguals), all studies converge in observing a switch cost.

Bultena et al. have argued that switch costs in sentence comprehension do not reflect language-wide control but instead reflect differences in the time it takes to retrieve a subjectively lower-frequency L2 word relative to a higher frequency L1 word (Bultena et al., 2015). To test this possibility, highly proficient Dutch–English bilinguals performed a self-paced reading study, which on critical trials contained an intra-sentential, full language switch at the determiner position. A switch cost was observed on the first content word following the code-switched determiner but only for L1-to-L2 switches. However, the self-paced reading paradigm lacks the temporal resolution necessary to disentangle what might have been earlier-acting control processes from later-acting sentence integration processes. In addition, the L1-to-L2 switch cost persevered across two words in the stimulus sentences, the first content word as well as the determiner that followed it. The authors note that this persistent cost reflects the incremental nature of sentence comprehension. This is at odds with the interpretation that longer L1–L2 switch costs in comprehension are the sole reflection of longer L2 word retrieval times.

Two of the studies cited earlier included both switch directions and both converge in finding switch costs in both directions (Litcofsky & Van Hell, 2017; Proverbio et al., 2004). These switch costs were observed in early time windows corresponding to the N1 and N400. These findings are problematic for the BIA+ because they suggest some language-wide control of activation even during early stages of lexical processing. What is striking about this parallel in findings is the significant differences in methodology across the two studies. In the study by Proverbio et al., participants were simultaneous interpreters while those recruited by Litcofsky and Van Hell were Spanish–English university students. In addition, the language switch in the Proverbio study was at the word-final position whereas Litcofsky and Van Hell implemented midsentence, full switches.

A third, eye-tracking study (Hoversten & Traxler, 2020) was designed to test the hypothesis that there is language-wide modulations of activation, thus resulting in differences in how accessible one language is over the other, what the authors refer to as the “zooming in hypothesis”. In the first experiment, Spanish–English bilinguals, who were more dominant in their L2, English, read L1 and L2 sentences across different language sessions. On critical trials, the sentences had a single code-switched word or a pseudoword embedded mid-sentence. If during comprehension, the current language being processed is more accessible, or what would be referred to as “zoomed into” then a word from the alternative language may be temporarily less accessible. In contrast, according to the BIA+ account, the earliest phases of lexical access are “blind” to language membership and thus any costs associated with switching should only emerge in eye-tracking measures that capture post-lexical access processing. The authors observed significant switch costs across eye-tracking measures capturing the early phases of lexical access, first fixation duration (FFD) and gaze duration (GD), as well as in measure capturing post-access integration processes, total reading time (TRT) and regression path duration.

In the second experiment, the critical, code-switched word (or pseudoword) only appeared in the parafoveal region of fixation. After the eyes crossed a boundary, the code-switched word or pseudoword would be replaced with the expected, same-language word. Such a unilingual context should allow for even stronger “zooming in” to the base language. If so, a code-switched word at the earliest phase of processing may be treated similar to a pseudoword. To test this hypothesis, the authors focused specifically on skipping rates, which reflect the earliest stages of lexical processing. If sufficient information from the parafoveal region is garnered early enough then a saccade to skip that word is programmed. If there is sufficient “zooming in” to the base language, then the orthographic information available from a code-switched word in the parafoveal region should be as uninformative as orthographic information from a pseudoword. Indeed, this is precisely what was observed. The reduction in skipping rates for code-switched words was similar to that for pseudowords.

The findings summarized above cannot be accommodated within an account that assumes early lexical access is impervious to language membership information. The first goal of the present study was to provide a direct test of the assumptions of the BIA+ by examining the processing of language switches in a natural reading task with eye tracking, which affords the temporal resolution necessary to discriminate between early, pre-lexical processes versus post-lexical processes. We also manipulated the cognate status of the switched word. If switch costs in comprehension are the sole reflection of word retrieval times, then a cost should only be observed for L1-to-L2 switches and this cost should be mitigated or eliminated when the switched word is a cognate. If, on the other hand, switch costs reflect language-wide modulations of activation then we should observe a cost in both directions. Observing the reverse, L2-to-L1 asymmetry would provide even more compelling evidence for language-wide modulations of activation. Prominent accounts of language control formulated based on language production, Green (1998) and Meuter and Allport (1999) postulate that one form of language control is through inhibition. Such accounts interpret L2-to-L1 asymmetrical costs as reflecting the greater amount of inhibition of the L1 that is exerted when processing in the L2, thus delaying switches back into the L1. A second goal of the present study was to test the assumption that early lexical access is similarly impervious to nonlinguistic cues. As mentioned previously, written comprehension typically obviates the need of a nonlinguistic cue. However, this does not mean that when such cues are present and informative that they cannot be used by the language system.

We are aware of only one published study that has directly tested the possibility that language selection can be informed by nonlinguistic cues (Fadlon et al., 2019). In that study, Spanish–English and Hebrew–English bilinguals read aloud paragraphs that were either mostly in the L1 or L2 and contained language-switched words that were either in a default font or cued by a red font color. The presence of a color-cued switch facilitated performance for both groups of bilinguals, specifically for L2-to-L1 switches. Color cues only facilitated L1-to-L2 switches for the Spanish–English bilinguals, and not the Hebrew–English bilinguals for whom the difference in writing script was sufficient of a cue to manage this switch direction. In the present study, we examine if such arbitrary color cues similarly facilitate comprehension of language switches using a natural reading task (eye-tracking) which does not require reading aloud and provides the temporal resolution necessary to identify the stage in processing in which color cues might modulate switch costs The present study also extends on Fadlon et al. in its examination of full, inter-sentential switches.

All stimuli data and analyses are available at https://osf.io/m4ay2/.

## 3. Methods

### 3.1. Participants

Participants were 51 Spanish–English bilinguals recruited from the University of Texas at El Paso. With 30 items per condition that allowed for 1,530 observations per condition giving adequate power to detect a small to medium effect size in mixed model analyses (see Brysbeart & Stevens, 2018). Responses on the English Spanish Proficiency and Dominance Assessment (ESPADA) (Francis & Strobach, 2013) indicated that participants had acquired Spanish on average at an earlier age than English. Their composite self-assessed proficiency rating for speaking, speech comprehension, reading and writing was significantly higher for English than Spanish, *t*(50) = 2.34, *p* < .05 (see Table 1). However, this difference was largely due to the higher rating for English writing. The ratings in all other domains, though numerically higher in English, were not significantly different from Spanish.

### 3.2. Picture vocabulary language proficiency

We used the picture naming vocabulary subtest of English and Spanish proficiency of the *Woodcock-Muñoz Language Survey Revised* (WMLS-R) (Muñoz-Sandoval, Ruef, & Alvarado, 2005) as an objective measure of language proficiency for 36 of the participants and the *Multilingual Naming Test* (MINT SPRINT) (Garcia & Gollan, 2022) for 15 of the remaining participants^1^. Average scores on both assessments were numerically higher in English than in Spanish (see Table 1). To classify participants’ dominance, we calculated the standard deviation in difference scores between the Spanish and English versions of each test separately. Participants whose difference score was greater than the 0.5 standard deviation were designated as dominant in the language of higher scored test; all others were designated as balanced. Based on this, metric 25 participants were English-dominant and 26 were balanced.

### 3.3. Stimuli

#### 3.3.1. Critical words

Critical words consisted of 120 Spanish–English translation pairs; 60 cognate nouns, and they were matched with 60 noncognate nouns based on CELEX word frequency and length in English (CELEX, Kerman, Piepenbrok, Baayan, & van Rijn, 1995) (see Table 2). To obtain an objective measure of the degree of orthographic form overlap of the cognates, we calculated the orthographic similarity ratio developed by Van Orden (1987). This measure includes a consideration of the number of single shared letters, the number of pairs of letters shared in forward and reverse order, and whether the first or last letters of the word pair are shared or not. For Spanish stimulus sentences, we used the Spanish translations of the English noncognate words. Cognates and matched controls were rotated through all possible conditions across four experimental running lists of 120 sentences using a Latin square design.

#### 3.3.2. Sentence stimuli

For each critical word, four sentences were created, one entirely in English, one entirely in Spanish, one with a language switch from English to Spanish, and one with a switch from Spanish to English. Sentences were written such that the meaning of the target word was not strongly biased (see Table 2). We used a cloze norming procedure to ensure that sentences did not strongly bias the critical words by presenting a sample of 15 highly proficient bilinguals with the entire sentence except the critical word. For 13 of these sentences, more than 30% of the respondents provided the critical word and these sentences were edited accordingly. For language-switched sentences, the critical cognate and noncognate nouns were the switch point. The switch always occurred in mid-sentence.

### 3.4. Apparatus

Eye movement data were obtained by using an Eye-Link 1000 tower-mounted system (SR-Research). Stimuli were presented binocularly, and eye movements were recorded from participants’ dominant eye. The right eye was used as default if the participant did not know which one of their eyes was the most dominant. Sentences were displayed on a 22-inch Samsung monitor. Participants were seated with a chin rest positioned 60 cm from a computer monitor. Sentences were presented in black 20-point Times New Roman Font on a white background using SR Research Experiment Builder. Sentences were displayed on the center of the screen.

### 3.5. Procedure

Before testing, participants completed informed consent. Next, participants completed the four subtests of the WMLS-R and then were accompanied to a private testing room and asked to sit in front of the eye-tracking display monitor, placing their chin on a chin rest. Next a nine-point calibration procedure was performed. Participants were then told that they would be presented with sentences in either English or Spanish and that some would contain a language switch. They were also told that true–false questions would follow some sentences.

Each trial started with a drift correction and then a fixation point appeared on the screen and participants were to press the spacebar to initiate a trial. Each sentence was presented one at a time in the center of the computer screen. Sentences from the four different conditions were intermixed through random selection. To ensure that participants were reading for comprehension, the participants were presented with a true/false question, after every 20 sentences.

After the sentence, comprehension task participants completed the ESPADA self-report survey (Francis & Strobach, 2013). Once participants had completed the survey, they were debriefed, thanked for their participation, granted credit and dismissed. The entire experiment took no more than 2 hours.

### 3.6. Data treatment and analyses

#### 3.6.1. Data trimming

All fixations that were shorter than 100 ms or longer than 2000 ms were removed from the data files and not included in the analyses. Any FFDs, GDs and TRTs that were longer or shorter than 2.5 standard deviations of the participant’s overall mean were removed, resulting in removal of 1.44% of the data.

#### 3.6.2. Data analyses

Data were extracted from a predefined interest area, which consisted of the critical cognate and noncognate words, which on switch trials, were the first word of the switch. We extracted the FFD, GD, Skip Rates and TRT. FFD is the duration in milliseconds of the first fixation made within an interest area. GD is the sum of the durations of all fixations made in an interest area before the eyes move forward (to the right) in the text. Skip rates is the percentage of times that the critical word was skipped. These three measures are assumed to tap into processes of lexical retrieval, from initial spread of activation to retrieval (Rayner 1998, 2009). TRT is the sum of the durations of all fixations made in an interest area, including regressions back to the interest area. It is assumed to reflect post-access processes, such as integration of the word into a representation of the clause or sentence (Rayner 1998, 2009).

#### 3.6.3. Statistical analyses

All dependent measures, except skipping, were log transformed. The data were analyzed through linear mixed-effects (LME) models (Gallucci, 2019) using the Jamovi statistical package (2021) which provides a drag-and-drop graphical programming environment based on the R (R Core Team, 2020) programming language (see Appendix B for all model summaries).

#### 3.6.4. Effects of switch condition and language

Although half of the sample was classified as balanced proficiency based on picture vocabulary tests, they are not valid indicators of reading skill and reading comprehension processes. As reflected in their responses on the ESPADA, participants read almost exclusively in English, suggesting that for bilinguals from this population English is the dominant language for reading. This dominance in English was also evident in reading time. We calculated the mean GD on control trials (language pure sentences with noncognates), this revealed that for participants designated as balanced proficiency all but two had shorter GDs in English than in Spanish. Thus, for the critical, linear mixed model analyses we designate English as the dominant language for all participants. We then report post hoc analyses performed on the English-dominant and balanced participants’ data separately.

To examine the effects of switch condition and direction of the switch we constructed models with two fixed factors: switch (switch, no switch) and language of the critical word (English, Spanish). All models included random intercept and slope adjustments by subjects for both fixed factors. A random intercept by item was also included, no other by-item adjustments were included as the factors were all item characteristics. We expected processing times to be significantly longer for switch relative to non-switch words for both English and Spanish critical words. Of critical importance for testing the assumptions of the BIA+ is that such switch costs would need to be manifest in aspects of the eye-tracking record the reflect pre-lexical access spread of activation, specifically: FFD, GD and skip rates. If language control involves active inhibition of the competing language then we would also expect larger switch costs when the critical word is in English (L2-to-L1 switches).

#### 3.6.5. Effects of switch condition and cognate status

To examine the effects of switch condition and direction of the switch we constructed models with two fixed factors: switch (switch, no switch) and cognate status of the critical word (cognate, noncognate). All models included random intercept and slope adjustments by subjects for both fixed factors. A random intercept by item was also included, no other by-item adjustments were included as the factors were all item characteristics. If switch costs in comprehension are not in fact switch costs but rather simply reflect differences in time to lexically retrieve a word, as assumed by the BIA+, then there should be an interaction between switch condition and cognate status such that the cost for L1-to-L2 switches are eliminated when the L2 switched word is a cognate. If, on the other hand, there is language-wide control we expect to observe a language-switch cost for cognate and noncognate trials. However, it is possible that for cognate trials the switch cost may not be evident in the earliest measures of the eye-tracking record such as FFD and skipping rates because there might not be sufficient orthographic information to indicate that the cognate is written in the competing language.

## 4. Results

### 4.1. Effects of switch condition and language

#### 4.1.1. Skip rates

Skipping rates were analyzed using a logistic generalized mixed-effects model. The analysis revealed a main effect of switch condition, with non-language-switched words being skipped more often (*M* = 0.12) than language-switched words (*M* = 0.09), *b* = 0.27, *SE* = 0.10, *z* = 2.61, *p* < .01. The interaction was marginally significant, *b* = 0.31, *SE* = 0.17, *z* = 1.77, *p* = .077. The nature of the interaction reflected a larger decrease in skipping rates for English critical words relative to Spanish critical words (see Table 3).

#### 4.1.2. First fixation duration

FFDs were significantly longer for language-switched words (*M* = 250) relative to same language words (*M* = 243), *b* = 0.0043, *SE* = 0.006, *t* = 2.92, *p* < .01 and longer for Spanish words relative to English words, *b* = 0.014, *SE* = 0.0047, *t* = 2.99, *p* < .01. The interaction between these two factors was significant, *b* = 0.025, *SE* = 0.008, *t* = 3.25, *p* < .01, reflecting a larger switch cost for Spanish-to-English switches than for English-to-Spanish switches (see Table 3).

#### 4.1.3. Gaze duration

GDs were significantly longer for language-switched words (M = 352) than non-language-switched words (M = 325), *b* = 0.036, *SE* = 0.007, *t* = 5.49, *p* < .001 as well as longer for Spanish words relative to English words. *b* = 0.07, *SE* = 0.01, *t* = 5.54, *p* < .001. The interaction between these two factors was significant, *b* = 0.05, *SE* = 0.01, *t* = 4.48, *p* < .001, reflecting a larger switch cost for Spanish-to-English switches than for English-to-Spanish switches (see Table 3).

#### 4.1.4. Total reading time

TRTs were significantly longer for language-switched words (M = 609) than non-language-switched words (M = 523), *b* = 0.07, *SE* = 0.009, *t* = 8.05, *p* < .001 as well as longer for Spanish words relative to English words, *b* = 0.09, *SE* = 0.02, *t* = 5.03, *p* < .001. The interaction between these two factors was significant, *b* = 0.06, *SE* = 0.01, *t* = 5.11, *p* < .001, reflecting a larger switch cost for Spanish-to-English switches than for English-to-Spanish switches (see Table 3).

#### 4.1.5. Spillover

Spillover durations were significantly longer for Spanish critical words (M = 258) relative to English critical words, *b* = 0.09, *SE* = 0.02, *t* = 5.03, *p* < .001. There were no other main effects or interactions (all *p*’s > .05).

#### 4.1.6. Post hoc analyses

We conducted post hoc analyses to determine if the nature of the language switch cost differed for participants whose scores on picture vocabulary reflected English dominance versus balanced proficiency. For these analyses we analyzed those aspects of the eye-tracking record most critical for testing the assumptions of the BIA + model, namely, skip rates, first fixation and GDs separately for English-dominant and balanced proficiency bilinguals. For the analyses on data from participants classified as English the interaction between switch condition and language remained highly significant for skip rates, *b* = 1.02, *SE* = 0.30, *z* = 3.38, *p* < .001; FFD, *b* = 0.04, *SE* = 0.01, *t* = 3.31, *p* < .001; and GD, *b* = 0.09, *SE* = 0.02, *t* = 5.00, *p* < .001. For participants classified as balanced proficiency there was no significant effect of switch condition or interaction with language of switch in either skipping rates or FFD. In the analyses on GD, there was a main effect of switch condition, *b* = 0.03, *SE* = 0.01, *t* = 2.86, *p* = .01, but the interaction with language was not significant, *b* = 0.025, *SE* = 0.02, *t* = 1.53, *p* = .13.

### 4.2. Effects of switch condition and cognate status

#### 4.2.1. Skip rates

The main effect of switch condition was significant, *b* = 0.28, *SE* = 0.10, *z* = 2.70, *p* < .01, there were no other main effects or interactions (all *p*’s > .05).

#### 4.2.2. First fixation duration

The main effect of switch condition was significant, *b* = 0.012, *SE* = 0.004, *t* = 2.88, *p* < .01, there were no other main effects or interactions (all p’s > .05).

#### 4.2.3. Gaze duration

The main effect of switch condition was significant, *b* = 0.035, *SE* = 0.007, *t* = 5.34, *p* < .001, there were no other main effects or interactions (all p’s > .05).

#### 4.2.4. Total reading time

The main effect of switch condition was significant, *b* = 0.072, *SE* = 0.009, *t* = 7.82, *p* < .001, there were no other main effects or interactions (all p’s > .05).

#### 4.2.5. Spillover

There were no significant main effect or interactions (all *p*’s > .05).

## 5. Discussion

The results of Experiment 1 revealed a significant cost in processing of language-switched words relative to same-language words throughout the eye-tracking record, even in measures tapping the earliest phases of lexical access, such as skipping rates. This suggest that at least by mid-sentence the nontarget language is less accessible than the language of the portion of the sentence being read. Furthermore, the switch cost was consistently asymmetrical, with greater costs when switching from the weaker language, Spanish into the stronger language, English. This suggests that the competing language was being controlled through inhibition. Because English was the more dominant language for reading, greater inhibitory control was required, thus, making it more costly to switch into that language. Furthermore, the fact that language-pure and language-switched sentences were mixed in the same experimental block likely increased the inhibitory control demands. Several production studies report a “mixing cost” in which naming latencies for non-switch trials in a mixed block are inflated relative to naming latencies in language pure blocks (see Declerck & Philipp, 2015 for a review). Not only is the observation of a general switch cost incompatible with the assumptions of the BIA+ the observation of a robust and consistent asymmetry in the *opposite* direction of what it would predict is particularly problematic for the model. As reviewed earlier previous comprehension studies have not consistently observed an asymmetrical switch back into the more dominant language. The post hoc analyses in the present experiment suggest that this inconsistency may be due in part to variation in language dominance profiles. It appears that an asymmetrical switch cost depends critically on there being a substantial relative difference in dominance.

We did not observe facilitated processing of cognates relative to noncognates. Previous research has shown that, when task demands accentuate differences in lexical form across cognate translations, cognate facilitation effects are eliminated and in some cases reversed (Dijkstra et al., 2010; Schwartz et al., 2007). For example, in a word naming task in which the full phonological form of words must be specified Schwartz et al. report a cost in performance for cognates with less similar phonology relative to noncognates. In the present experiment the frequent, mid-sentence switches at the point of a highly similar cognate likely resulted in a higher level of monitoring for language and which could have accentuated finer -grained distinctions in orthographic form between cognate translations. This could be responsible for the absence of a facilitation effect.

## 6. Experiment 2

The goals of Experiment 2 were twofold. First, we sought to replicate the switch cost observed in Experiment 1 with a completely different set of sentence stimuli. Second, and more crucially, we sought to test the hypothesis that nonlinguistic cues of language membership can be exploited by cognitive general control mechanisms when they are available. We used a published set of sentences with midway inter-sentential switches Litcofsky and Van Hell (2017). These sentences elicited ERP components reflecting a switch cost in a population of Spanish–English university student bilinguals who, similar to the population we sampled from, had for the most part acquired Spanish first, but then received significant schooling in English. The first forty trials did not contain any color cue, allowing us to replicate the switch cost observed in Experiment 1. This was followed by two additional blocks of trials. For one block all sentences contained a language switch and each language was paired with a particular font color. If the language system can exploit such cues we expected to observe a significant attenuation in the switch cost for this block relative to the first block. To control for potential spurious effects of reading color switched sentences a third block of trials consisted of language pure sentences, with a color switch midsentence. The order of these latter two blocks was counterbalanced across participants.

## 7. Methods

### 7.1. Participants

An original sample of 66 highly proficient Spanish–English bilinguals from the same population of Experiment 1 participated in the experiment. Data from four participants were not included in analyses due to insufficient proficiency in Spanish. Data from one participant were excluded due to a recording error, leaving a total analyzable sample of 61. With 30 items per condition this sample size was adequately powered to detect small to medium effect in mixed model analyses (Brysbaert & Stevens, 2018). Overall responses on the ESPADA (Francis & Strobach, 2013) were similar to those of Experiment 1. Once again participants acquired Spanish on average at an earlier age than English. Their composite self-assessed proficiency rating for speaking, speech comprehension, reading and writing was marginally significantly higher for English than Spanish, *t*(60) = 1.94, *p* = .056 (see Table 1).

### 7.2. Picture vocabulary language proficiency

We used the picture naming vocabulary subtest of English and Spanish proficiency of the *Woodcock-Muñoz Language Survey Revised* (WMLS-R) (Muñoz-Sandoval, Ruef, & Alvarado, 2005) as an objective measure of language proficiency. Using the same metric as in Experiment 1 to classify language dominance, 22 were English-dominant, 25 were balanced and 14 were Spanish-dominant.

### 7.3. Stimulus sentences

We selected 120 base sentences from Litcofsky and Van Hell (2017). Each base sentence had four versions, all English, all Spanish, switch from English to Spanish and switch from Spanish to English. For code-switched versions of the sentences, the switch was followed by at least three words and all words following the code switch remained in the same code-switched language. The critical switched words and controls were all nouns, and in the Spanish versions, none contained diacritical markings. All four versions of the 120 base sentences were rotated through three cue conditions: no color cue (switched and non-switched sentences), color cue (all switched sentences, with color cue mapped to each language) and color cue/no switch (all non-switched sentences with change in color font midsentence). To ensure that, no version of any base sentence appeared twice eight experimental running lists were created based on a Latin Square.

### 7.4. Color-cueing procedure

Each session was divided into three blocks. The first block served as a baseline, in which sentences did not have any language color cueing. The second and third blocks consisted of sentences written in blue and red colored fonts. For one of these blocks each color was consistently mapped to each language and all sentences contained a language switch, thus there was a color switch and language switch. For the other block sentences were all language pure, containing no language switch, but the font color switched. The order of these two colored font blocks was counterbalanced across participants.

#### 7.4.1. Apparatus

Same as in Experiment 1.

#### 7.4.2. Procedure

Same as in Experiment 1.

## 8. Data treatment and analyses

### 8.1. Data trimming

All fixations that were shorter than 100 ms were removed from the data files and not included in the analyses. Also, any fixations longer than 2000 ms were removed. Finally, any FFDs, GDs and TRTs that were longer or shorter than 2.5 standard deviations of the participant’s overall mean were removed, resulting in removal of 1.62% of the data.

### 8.2. Data analyses

Data were extracted from a predefined interest area, which consisted of the code-switched word and its matched control. Once again, we extracted skip rates, FFD, GD, spillover and TRT.

### 8.3. Statistical analyses

All dependent measures were log transformed, except skips. The data were analyzed through LME models and analyses on skipping rates were analyzed through generalized logistic mixed-effects models (Gallucci, 2019) using the Jamovi statistical package (2021) which provides a drag-and-drop graphical programming environment based on the R (R Core Team, 2020) programming language.

### 8.4. Fixed factors structure

We constructed two separate sets of models. The first set of models was to test if the observed asymmetrical switch cost of Experiment 1 would replicate with a completely different set of stimuli of Experiment 2. In those models, the fixed factors were switch condition (switch, non-switch) and language of critical word (English, Spanish). The second set of models were designed to test for the potential effects of color cue on language-switched trials. In those models, the fixed factors were switch condition (switch, non-switch) and color cue (cue, no cue). It should be noted that the design was not fully crossed, all non-switch sentences were from block 3 and were in a colored font, there was no “non-switch/no cue” condition.

As in Experiment 1, participants’ responses on the ESPADA and reading times reflected English dominance in reading. However, unlike Experiment 1, there were four participants whose picture vocabulary score difference and reading times reflected Spanish dominance. Thus, in the analyses assessing the potential asymmetrical switch cost from Spanish into English, data from these four participants were excluded.

### 8.5. Random factors structure

All models included random intercept and slope adjustments by subjects for all fixed factors. A random intercept by item was also included; no other by-item adjustments were included as the factors were all item characteristics.

## 9. Results

### 9.1. Effect of switch condition and language of switched word

#### 9.1.1. Skip rates

Skipping rates were significantly lower for critical words that were language-switched (*M* = 0.09) versus in the same language (*M* = 0.11), *b* = 0.55, *SE* = 0.17, *z* = 3.30, *p* < .001. Skipping rates were significantly lower for Spanish critical words (*M* = 0.09) relative to English critical words (*M* = 0.11), *b* = 0.56, *SE* = 0.16, *z* = 3.51, *p* < .001. There was no significant interaction (*p* > .05).

#### 9.1.2. First fixation duration

FFDs were significantly longer for language-switched words (*M* = 267) relative to same language words (*M* = 249), *b* = 0.02, *SE* = 0.0045, *t* = 5.07, *p* < .001. FFDs were also significantly longer for Spanish critical words (*M* = 263) relative to English critical words (*M* = 252), *b* = 0.02, *SE* = 0.004, *t* = 5.07, *p* < .001. The interaction between these two factors was significant, *b* = 0.03, *SE* = 0.0080, *t* = 3.82, *p* < .001, reflecting a larger switch cost for Spanish-to-English switches than for English-to-Spanish switches (see Table 3).

#### 9.1.3. Gaze duration

GDs were significantly longer for language-switched words (*M* = 383) relative to same language words (*M* = 329), *b* = 0.06, *SE* = 0.0057, *t* = 10.33, *p* < .001. GDs were also significantly longer for Spanish critical words (*M* = 394) than English critical words (*M* = 318), *b* = 0.07, *SE* = 0.013, *t* = 5.38, *p* < .001. The interaction between these two factors was significant, *b* = 0.05, *SE* = 0.010, *t* = 4.69, *p* < .001, reflecting a larger switch cost for Spanish-to-English switches than for English-to-Spanish switches (see Table 3).

#### 9.1.4. Total reading time

TRTs were significantly longer for language-switched words (*M* = 554) relative to same-language words (*M* = 437), *b* = 0.10, *SE* = 0.0084, *t* = 11.79, *p* < .001. TRTs were also significantly longer for Spanish critical words (*M* = 564) relative to English critical words (*M* = 426), *b* = 0.10, *SE* = 0.018, *t* = 5.68, *p* < .001. The interaction between these two factors was significant, *b* = 0.036, *SE* = 0.011, *t* = 3.10, *p* < .01, reflecting a larger switch cost for Spanish-to-English switches than for English-to-Spanish switches (see Table 3).

#### 9.1.5. Spillover

There were no significant main effects or interactions (all *p*’s > .05).

### 9.2. Effect of color cue on language switching

#### 9.2.1. Skip rates

Skipping rates were significantly lower for language-switched critical words (*M* = 0.09) than same language critical words (*M* = 0.09), *b* = 0.57, *SE* = 0.17, *z* = 3.34, *p* < 0.001. There were no other main effects or interactions (all *p*’s > .05).

#### 9.2.2. First fixation duration

FFDs were significantly longer for language-switched critical words (*M* = 269) than same language critical words (*M* = 249), *b* = 0.03, *SE* = 0.0047, *z* = 5.57, *p* < .001. This was qualified by an interaction with color cue, *b* = 0.02, *SE* = 0.0085, *t* = 2.57, *p* < .05, reflecting a smaller switch cost for color-cued sentences relative to non-cued sentences (see Table 4).

#### 9.2.3. Gaze duration

GDs were significantly longer for language-switched critical words (*M* = 277) than same language critical words (*M* = 261), *b* = 0.03, *SE* = 0.005, *t* = 5.57, *p* < .001. There was a main effect of color cue, *b* = 0.04, *SE* = 0.013, *t* = 2.99, *p* < .01, reflecting longer durations for non-color-cued words (*M* = 380) than color-cued words (*M* = 344). This was qualified by a significant interaction with language switch condition, *b* = 0.05, *SE* = 0.011, *t* = 4.27, *p* < .001, reflecting a smaller switch cost for color-cued sentences relative to non-cued sentences (see Table 4).

#### 9.2.4. Total reading time

TRTs were significantly longer for language-switched critical words (*M* = 641) than same language critical words (*M* = 510), 0.07, *SE* = 0.018, *t* = 3.94, *p* < .001. There was a main effect of color cue, *b* = 0.07, *SE* = 0.02, *t* = 3.94, *p* < .001, reflecting longer durations for non-color-cued words (*M* = 553) than color-cued words (*M* = 467). This was qualified by a significant interaction with language switch condition, *b* = 0.06, *SE* = 0.01, *t* = 4.89, *p* < .001, a smaller switch cost for color-cued sentences relative to non-cued sentences (see Table 3).

#### 9.2.5. Spillover

There were no significant main effects or interactions (all *p*’s > .05).

## 10. Discussion

We succeeded in replicating a language switch cost with a completely different set of stimulus sentences. As in Experiment 1, an asymmetrical switch cost that was larger in the L2-to-L1 direction was observed throughout the eye-tracking record, in skipping rate, first fixation and GDs and TRTs. This asymmetry is completely opposite of what would be predicted if switch costs in comprehension were solely a function of relative differences in the time it takes to retrieve an L1 versus L2 switched word. Instead, the direction of this asymmetry suggests a retroactive inhibition control mechanism at work, in which it takes longer to overcome the greater amount of inhibition needed to suppress L1 words relative to L2 words.

In terms of nonlinguistic cues, the results of Experiment 2 support the hypothesis that the language system can in fact exploit such cues when they are present. The magnitude of the language-switch cost was significantly reduced for color-cued sentences than noncolor-cued sentences. This was observed in FFD, GD and TRT. We now turn to a general discussion of the implications of the results of these two experiments for current models of the bilingual lexicon.

## 11. General discussion

One goal of the present study was to test the assumption of the BIA+ model (Dijkstra & van Heuven, 2002) that there is no top-down, language-wide inhibitory control operating within the mental lexicon. The BIA+ diverged fundamentally from its predecessor model, the BIA (Dijkstra & van Heuven, 1998), regarding factors that affect the activation level of lexical representations across the target and nontarget languages during language processing. In the BIA model, there existed language nodes that exert top-down, inhibitory control of the nontarget language, allowing representations within one language to be more accessible than those in the alternative language. In the BIA+, language-wide inhibitory control is handled outside of the lexicon, in a separate task-decision system. The key implication of this architectural change is that language membership information has no direct effect on the processes of lexical activation that precede lexical access. In the present study, we tested this assumption across two experiments through a language-switching paradigm.

If there is no top-down inhibitory control of language within the lexicon, then we should not observe an overall processing time cost for language-switched words versus non-switched words in aspects of the eye-tracking record that capture pre-lexical access processes. Instead, we should only observe inflated processing times for L1-to-L2 switches relative to the reverse direction, and this cost would solely reflect the longer time it takes to retrieve subjectively lower frequency L2 words. Results from both experiments falsify this prediction. Not only was there an overall language switch cost observed across both experiments, the magnitude of the cost was asymmetrical, with a greater cost when switching back into the more dominant language, English. This is the opposite direction of what would be predicted by the BIA+. Furthermore, the asymmetry was observed across both experiments in FFD and GD, which capture lexical access processes before completion of lexical retrieval. The architecture of the BIA+ cannot accommodate these findings.

The L2-to-L1 asymmetry is consistent with inhibitory control processes in which the L1 has to be more strongly inhibited in the interest of processing in the weaker L2. As a result, switching back into the L1 requires overcoming a greater amount of suppression. This L2-to-L1 asymmetry has been widely reported in production studies. Yet, in comprehension studies, an asymmetry is rarely observed, and when it is, it is in the L1-to-L2 direction. For example, Bultena et al. (2015) observed a switch cost only in the L1-to-L2 direction in their self-paced reading study. However, self-paced reading times cannot discriminate between early versus later occurring lexical activation processes. The L1-to-L2 asymmetry observed by Bultena et al. may be a reflection of later-stage, post-lexical access updating of the mental representation of the sentences. Recent EEG research demonstrates that there is a greater cognitive demand in integrating L2 words into L1-based semantic representations. According to Litcofsky and Van Hell (2017) when bilingual read sentences in their more dominant L1, integrating a code-switched L2 word requires an entire restructuring of the mental representation of the sentence. This may be the mechanism responsible for the asymmetry observed by Bultena et al. Finally, the asymmetrical switch cost of the present study might have been particularly robust due to the mixed nature of the experimental blocks. Findings from production studies suggest that the demands of inhibitory control of the dominant language are increased in blocks in which code-switched and language-pure trials are intermixed relative to when they are blocked.

The present findings demonstrate that the observation of an asymmetrical, L2–L1 switch cost depends critically on there being a sufficient relative difference in language proficiency. In Experiment 1, nearly half of the participants were classified as having balanced proficiency based on standardized picture vocabulary measures. Post hoc analyses conducted on the data from these participants revealed a switch cost in GD that did not interact with the language of the switched words. In other words, the L2–L1 asymmetry reflecting stronger inhibition processes was not evident for balanced bilinguals. The implication is that when proficiency is balanced, language control is also exerted, as reflected by a general switch cost; however, one language does not require significantly more inhibitory control than the other.

The observed switch costs across Experiments 1 and 2 are consistent with more recent studies on the comprehension of language-switched sentences that have used measures with adequate temporal resolution to tap into the earliest stages of lexical processing such as eye tracking and Electroencephalography (EEG). As reviewed earlier, Hoversten and Traxler (2020) observed a language switch cost in aspects of the eye-tracking record capturing early phase processing, such as skipping rates and FFD. The reader will recall that in that study code-switched words were responded to similarly to pseudowords, suggesting that the overall activation level of the nontarget language was significantly suppressed. The authors interpret their findings as reflecting “partially selective access” which is consistent with a “zooming in hypothesis”. According to this hypothesis, language selectivity versus non-selectivity is not an all-or-none dichotomy, instead language-wide activation levels change dynamically in response to incoming language stimuli. In this way, continuous input from language “A” allows the global activation of that language to be higher than language “B”. This hypothesis was originally proposed by Elston-Güttler et al. (e.g., Elston-Güttler et al., 2005; Paulman et al., 2006), who observed that when bilinguals first viewed a film in the weaker L2 the effects of the L1 on L2 sentence processing were eliminated in the block of trials immediately following the film.

Neuroimaging studies on isolated word recognition tasks provide converging evidence for the “zooming in hypothesis” (Hoversten et al., 2015; Peeters et al., 2019; Rodriguez-Fornells et al., 2002) For example, in a go/no-go fMRI/ERP study (Rodriguez-Fornells et al., 2002) highly proficient speakers of Spanish and Catalan were asked to selectively respond to Spanish words while ignoring Catalan words and nonwords. Only evoked potentials (N400) in response to Spanish words were modulated by word frequency, indicating those words had been lexically accessed. In contrast, no such modulation was observed for Catalan words, despite the fact that participants were able to quickly and accurately identify the language of those words. Complimenting these findings is fMRI research demonstrating that the language-control network in the brain is also highly active when bilinguals perform lexical decisions on word stimuli that have a high-degree of cross-language overlap (Peeters et al., 2019).

The language nodes of the original BIA (Dijkstra & van Heuven, 1998) do in fact allow for language-wide modulations of activation, exerted top-down from the nodes to the words of the competing language in the lexicon. Because the L1 node collects greater activation from its higher frequency L1 words, it exerts more inhibition on the L2 compared to the L2 node, which collects less activation from its lower frequency words. This type of language control is sustained, accumulated inhibition of the competing language over time and aligns perfectly with “zooming in”.

A second goal of the present study was to test the assumption, shared across both the BIA and BIA+ models, that lexical activation dynamics within the bilingual lexicon are unaffected by nonlinguistic information or cues. We included a block **of** language-switched sentences in Experiment 2 in which language was cued by font color. According to both the BIA and BIA+ models, nonlinguistic information only affects processing post-lexical access. Therefore, these models would predict that any effects of language color cueing should only be observed in measures tapping later processing, namely, TRT. Importantly, no such effects should be observed in skipping rates or first fixation or GDs. In contrast to these predictions, we observed effects of color-cued switches in first fixation and GD analyses. It is noteworthy that color cueing did not have an impact on skipping rates. This suggests that perceiving the color in the parafoveal region, without sufficient orthographic input, was not an effective cue for modulating the earliest phases of lexical access. The observed benefit of color cueing is consistent with the findings of Fadlon et al. (2019) in which color cueing reduced nontarget language intrusions during paragraph reading. The color cue benefit was consistently observed for Spanish–English bilinguals across low- and high-demand tasks. In contrast, color cueing affected performance of Hebrew–English bilinguals only in high-demand tasks. Therefore, the effectiveness of an arbitrary, nonlinguistic cue is shaped fundamentally by the additional information it provides. When effective natural cues are in place, such as those present in distinctive writing systems, more arbitrary cues are less likely to be attended to.

These findings suggest that the initial phases of lexical access are not completely insular from cognitive general control processes. Only a few bilingual comprehension studies have directly tested whether cognitive general control processes can directly modulate the relative activation levels of words in a target versus nontarget language (e.g., Dijkstra, De Bruijn et al., 2000; Dijkstra, Timmermans & Schriefers, 2000). The general approach in these studies has been to compare performance in recognizing interlingual homographs in lexical decision across different task parameters, such as explicitness and nature of instructions. In an L2, English lexical decision experiment, Dutch–English bilinguals were explicitly told that they would encounter interlingual homographs, that should be responded to with a “yes” response (Dijkstra, De Bruijn et al., 2000). However, knowing ahead of time that these tricky trials existed in the stimulus list did not result in interlingual homograph inhibition effects. Such effects only emerged when Dutch-exclusive words, requiring a “no” response were included in the stimulus list. Thus, knowing the nature of the task before hand did not allow bilinguals to differentially suppress the L1 in anticipation of the tricky trials. In another study (Dijkstra, Timmermans & Schriefers, 2000), the nature of the instructions was manipulated, interlingual homographs were presented in either a language general task (respond “yes” if it’s a word in either language) or language exclusive task (respond “yes” only to words in the target language). In the latter case, optimal performance would result from completely inhibiting or turning off the nontarget language. The results showed that bilinguals were unable to selectively turn off one language.

At first, it may seem these published studies are inconsistent with the present results and those of Fadlon et al. (2019). However, the pattern of findings across studies can be understood by considering fundamental differences in the nature of the critical stimuli and the prevalence of the cue. In the studies by Dijsktra et al. reported above, the critical stimuli were completely language ambiguous, being interlingual homographs. Therefore, the “cue” adopted in these studies, explicitness of instruction or the nature of instructions, does not allow for language membership disambiguation. In addition, the “cue” of instructions, unlike a color cue, is not a cue that can be directly perceived by the senses. A key future direction is a systematic examination and comparison of the effects of different types of cues on the relative accessibility of words in the nontarget language. Our working hypothesis is that cognitive general control processes can be engaged early on in processing when there are informative, prevalent and directly perceptible cues.

## 12. Conclusions

In conclusion, in the present study, we tested two critical assumptions of the BIA+ (Dijkstra & van Heuven, 2002): (1) that there is no language-wide control of activation during written comprehension and (2) that initial lexical access processes are impervious to nonlinguistic cues. The observation of language switch costs and in particular the fact that they were greater in the L2-to-L1 direction across two experiments, refutes the first assumption, providing strong evidence that language-wide activation levels are modulated during reading comprehension. To accommodate this finding, the BIA+ architecture should incorporate the language nodes of the original BIA (Dijkstra & van Heuven, 1998). The observation of reduced switch costs in the presence of a color cue in Experiment 2 refutes the second assumption and provides evidence that the language system can exploit a variety of environmental cues. To accommodate this finding, the architecture of the BIA+ should be modified in such a way that there are bidirectional links between the lexicon and the task schema system.

## Supplementary Material

Experiment 1 language proficiency data

Experiment 2 Language proficiency data

Experiment 2 raw data

Experiment1 raw data

Experiment 1 stimuli

LME models

## Figures and Tables

**Table 1.** Language proficiency characteristics of bilingual participants across Experiments 1 and 2 (standard deviations in parentheses)

Experiment 1		
	English	Spanish
Age of acquisition^a^	6.3	2.4
	(3.4)	(2.0)
Self-rated reading proficiency^b^	8.4	7.9
	(1.4)	(1.5)
Writing	8.4	7.0
	(1.2)	(1.7)
Speaking	8.7	8.5
	(1.2)	(1.5)
Speech comprehension	9.1	8.8
	(1.2)	(1.3)
Picture vocabulary WMLS-R^c^	84.6	80.0
	(12.0)	(7.6)
Picture vocabulary MintSprint^d^	64.1	52.3
	(9.9)	(16.1)
Mean gaze duration	262	355
	(159)	(276)
Experiment 2		
	English	Spanish
Age of acquisition^a^	6.0	2.1
	(4.0)	(2.2)
Self-rated reading proficiency^b^	8.4	7.9
	(1.3)	(1.7)
Writing	8.2	7.2
	(1.4)	(1.9)
Speaking	8.5	8.4
	(1.3)	(1.7)
Speech comprehension	8.8	8.7
	(1.3)	(1.7)
Mean gaze duration	259.1	347.0
	(171)	(262)

a In years.

b On a scale of 1–10.

c Averaged across 36 participants.

d Averaged across 15 participants.

**Table 2.** Lexical characteristics of critical cognate and noncognate control words and example sentences used in Experiment 1

	English frequency^a^	English length	Spanish length	Cross-language orthographic similarity ratio^b^
Cognate *train/tren*	16.79	5.97	6.50	0.63
example sentence stimuli	The two sisters took the **train** to visit their parents.			
	Las dos hermanas tomaron el **tren** para visitar a sus padres.			
	The two sisters took the **tren** para visitar a sus padres.			
	Las dos hermanas tomaron el **tren** to visit their parents.			
NonCognate *piscina/pool*	17.91	5.77	6.68	0.15
example sentence stimuli	The brothers went to the **pool** to swim with friends.			
	Los hermanos fueron a la **piscina** a nadar con sus amigos.			
	The brothers went to the **piscina** a nadar con sus amigos.			
	Los hermanos fueron a la **pool** to swim with friends.			

a Occurrences per 1 million words, derived from SubtlexUs.

b Van Orden (1987).

**Table 3.** Mean percent skipping rates, first fixation and gaze duration, spillover and total reading time in milliseconds as a function of switch condition and language of critical word for Experiment 1 (standard deviations in parentheses) and Experiment 2

Experiment 1				
	English		Spanish	
	Switch	Non-switch	Switch	Non-switch
Skip rate	0.094	0.13	0.096	0.1
	(0.29)	(0.34)	(0.29)	(0.3)
	***−.036***		***−.004***	
First fixation duration	251	233	249	253
	(99.9)	(81.2)	(90.4)	(105)
	***+18***		***−4***	
Gaze duration	325	276	378	373
	(172)	(130)	(274)	(260)
	***+49***		***+5***	
Total reading time	551	431	666	614
	(400)	(300)	(541)	(493)
	***+120***		***+52***	
Spillover	257	244	262	256
	(123)	(96.7)	(119)	(113)
	***+13***		***+6***	
Experiment 2				
	English		Spanish	
	Switch	Non-switch	Switch	Non-switch
Skip rate	0.097	0.12	0.087	0.099
	(0.29)	(0.33)	(0.28)	(0.30)
	***−0.023***		***−0.012***	
First fixation duration	266	239	267	259
	(129)	(95.5)	(158)	(117)
	***+27***		***+8***	
Gaze duration	353	283	412	375
	(208)	(151)	(297)	(261)
	***+70***		***+37***	
Total reading time	486	366	621	507
	(328)	(230)	(485)	(390)
	***+120***		***+114***	
Spillover	239	233	244	242
	(89.5)	(87.8)	(107)	(95.8)
	***+6***		***+2***	

**Table 4.** Means and standard deviations of reading time (in milliseconds) by color-cue and switch condition for Experiment 2

	Color cue		No color cue	
	Switch	No switch	Switch	No switch
Skip rate^a^	0.09	0.11	0.10	0.11
	(0.29)	(0.31)	(0.29)	(0.32)
	***−0.02***		***−0.01***	
First fixation duration	261	250	277	246
	(127)	(111)	(172)	(100)
	***+11***		***+31***	
Gaze duration	361	326	425	335
	(240)	(221)	(286)	(212)
	***+35***		***+90***	
Total reading time	510	423	641	465
	(393)	(314)	(455)	(354)
	***+87***		***+176***	
Spillover	242	237	242	239
	(92)	(89)	(114)	(98)
	***+5***		***+3***	

a Percent probability.

## Highlights

- We provide evidence that the nontarget language is inhibited during reading
- Nonlinguistic color cues facilitate language control processes
- We discuss implications for models of the bilingual lexicon

## Footnotes

1 A shorter assessment was used due to experimental running timing restrictions.
