# Supplementary material for: The recruitment of global language inhibitory control and cognitive-general control mechanisms in comprehending language switches: Evidence from eye movements
Source: Biling (Camb Engl). Author manuscript; Available in PMC 2026 Mar 1. (PMC12180542; doi:10.1017/S1366728924000567)
Supplement: LME models [file NIHMS2051699-supplement-LME_models.pdf]

Appendix A

Summary of Linear Mixed Effects Models

Experiment 1								
~ 1 + switchcond + Language + switchcond:Language + (1 | itemnumber) + (1 + switchcond + Language | subject)								
	Skip				FFD			
Model effects	b	SE	t, z value	Variance	b	SE	t, z value	Variance
Fixed effects								
Language	0.133	0.120	1.11	1.51e-5	0.014	0.0047	2.99	2.78e-4
Switch condition	0.272	0.104	2.61	8.40e-5	0.0125	0.0042	2.92	1.63e-4
Language X Switch Condition	0.308	0.17	1.77		0.0252	0.0078	3.24	
Intercept	2.51	0.147	17.08		2.36	0.0068	346.76	
Random effects								
Subject variance				0.888				0.00215
Item variance				0.211				1.07e-4
Residual variance				1.000				0.02100
	GD				Spillover			
Model effects	b	SE	t, z value	Variance	b	SE	t, z value	Variance
Fixed effects								
Language	0.068	0.012	5.54	0.004	0.014	0.0057	1.702	1.67e-5
Switch condition	0.036	0.007	5.49	7.83e-4	0.011	0.0064	2.464	7.15e-4
Language X Switch Condition	0.048	0.011	4.48		0.009	0.0100	0.909	
Intercept	2.46	0.01	241.39		2.370	0.0082	290.630	
Random effects								
Subject variance				0.004				0.003
Item variance				0.003				3.91e-4
Residual variance				0.040				0.024
	TRT							
Model effects	b	SE	t, z value	Variance				
Fixed effects								
Language	0.09	0.019	5.03	0.009				
Switch condition	0.07	0.009	8.05	0.002				
Language X Switch Condition	0.06	0.013	5.11					
Intercept	2.65	0.018	151.27					
Random effects								
Subject variance				0.013				
Item variance				0.009				
Residual variance				0.056				

Experiment 1								
~ 1 + switchcond + Cognate status + switchcond:cognate status + (1 | itemnumber) + (1 + switchcond + cognate status | subject)								
	Skip				FFD			
Model effects	b	SE	t, z value	Variance	b	SE	t, z value	Variance
Fixed effects								
Cognate Status	0.017	0.123	0.139	0.036	0.001	0.004	0.286	1.55e-4
Switch condition	0.280	0.104	2.704	0.001	0.012	0.004	2.882	6.15e-5
Cognate Status X Switch Condition	0.010	1.010	0.059		0.003	0.008	0.401	
Intercept	2.516	0.147	17.069		2.362	0.007	345.212	
Subject variance				0.888				0.002
Item variance				0.217				1.49e-4
Residual variance				1.00				0.021
	GD				Spillover			
Model effects	b	SE	t, z value	Variance	b	SE	t, z value	Variance

Fixed effects								
Cognate Status	0.008	0.010	0.819	2.06e-4	0.002	0.006	0.343	1.73e-4
Switch condition	0.036	0.007	5.336	8.11e-4	0.010	0.006	1.624	7.02e-4
Cognate Status X Switch Condition	7.53e-4	0.011	0.070		0.017	0.010	1.658	
Intercept	2.464	0.011	0234.058		2.370	0.008	289.101	
Random effects								
Subject variance				0.004				0.003
Item variance				0.004				4.31e-4
Residual variance				0.041				0.024

	TRT			
Model effects	b	SE	t, z value	Variance
Fixed effects				
Cognate Status	0.003	0.014	0.195	7.59e-4
Switch condition	0.072	0.009	7.824	0.002
Cognate Status X Switch Condition	0.014	0.013	1.046	
Intercept	2.651	0.018	148.832	
Random effects				
Subject variance				0.013
Item variance				0.011
Residual variance				0.058

Experiment 2								
~ 1 + switchcond + Language + switchcond:Language + (1 | itemnumber) + (1 + switchcond + Language | subject)								
	Skip				FFD			
Model effects	b	SE	t, z value	Variance	b	SE	t, z value	Variance
Fixed effects								
Language	0.558	0.159	3.51	0.120	0.014	0.006	2.43	5.31e-4
Switch condition	0.552	0.168	3.30	0.307	0.022	0.004	5.07	2.37e-4
Language X Switch Condition	0.093	1.097	0.48		0.031	0.008	3.82	
Intercept	3.359	0.266	12.63		2.375	0.006	368.61	
Subject variance				3.634				0.002
Item variance				0.169				3.96e-4
Residual variance				1.00				0.025
	GD				Spillover			
Model effects	b	SE	t, z value	Variance	b	SE	t, z value	Variance
Fixed effects								
Language	0.071	0.013	5.38	0.003				
Switch condition	0.059	0.006	10.33	4.05e-4				
Language X Switch Condition	0.048	0.010	4.69					
Intercept	2.480	0.010	256.55					
Random effects								
Subject variance				0.004				
Item variance				0.006				
Residual variance				0.041				
	TRT							
Model effects	b	SE	t, z value	Variance				
Fixed effects								
Language	0.010	0.018	5.68	0.006				
Switch condition	0.099	0.008	11.79	0.002				
Language X Switch Condition	0.036	0.011	3.10					
Intercept	2.596	0.013	193.61					
Random effects								

Subject variance				0.008
Item variance				0.011
Residual variance				0.051

Experiment 2								
~ 1 + switchcond + color cue + switchcond:colorcue+ (1 | itemnumber) + (1 + switchcond + colorcue | subject)								
	Skip				FFD			
Model effects	b	SE	t, z value	Variance	b	SE	t, z value	Variance
Fixed effects								
Color Cue	0.299	0.203	1.47	0.533	0.009	0.006	1.51	4.19e-4
Switch condition	0.569	0.171	3.34	0.316	0.026	0.005	5.57	2.47e-4
Color Cue X Switch Condition	0.046	0.209	0.22		0.022	0.009	2.57	
Intercept	3.426	0.275	12.46		2.376	0.007	362.76	
Random effects								
Subject variance				3.835				0.002
Item variance				0.208				4.34e-4
Residual variance				1.00				0.025
	GD				Spillover			
Model effects	b	SE	t, z value	Variance	b	SE	t, z value	Variance
Fixed effects								
Color Cue	0.040	0.013	2.99	0.001	0.003	0.007	0.39	4.82e-4
Switch condition	0.067	0.006	11.03	4.45e-4	0.004	0.006	0.71	1.91e-4
Color Cue X Switch Condition	0.046	0.011	4.27		0.014	0.012	1.21	
Intercept	2.486	0.010	251.89		2.347	0.007	336.20	
Random effects								
Subject variance				0.003				0.002
Item variance				0.007				4.02e-4
Residual variance				0.041				0.021
	TRT							
Model effects	b	SE	t, z value	Variance				
Fixed effects								
Color Cue	0.071	0.018	3.94	0.004				
Switch condition	0.108	0.009	12.28	0.002				
Color Cue X Switch Condition	0.060	0.012	4.89					
Intercept	2.608	0.014	187.86					
Random effects								
Subject variance				0.008				
Item variance				0.012				
Residual variance				0.052				
